# Body Size and Risk of Death During the 1918 Influenza Pandemic in Alaska

**DOI:** 10.1002/ajhb.70151

**Published:** 2025-10-01

**Authors:** Taylor P. van Doren, Lauren E. Steele, Emma Tinker‐Fortel, Lisa Sattenspiel

**Affiliations:** ^1^ Institute for Circumpolar Health Studies University of Alaska Anchorage Anchorage Alaska USA; ^2^ Department of Sociology & Anthropology High Point University High Point North Carolina USA; ^3^ School of Chemistry & Molecular Biosciences, Faculty of Science University of Queensland Brisbane Australia; ^4^ Department of Anthropology University of Missouri Columbia Missouri USA

**Keywords:** 1918 influenza pandemic, BMI, developmental origins of health and disease, historical epidemiology, syndemics

## Abstract

In the aftermath of the 2009 H1N1 influenza A pandemic, research revealed the relationship between body size and the severity of influenza outcomes. However, there is little data available on body size in historical populations; therefore, the relationship between body size and 1918 influenza pandemic outcomes is virtually unknown. Alaskan death records from the Alaska Bureau of Vital Statistics with recorded height and mass at death (*n* = 2724) were analyzed to illuminate this relationship during both the pandemic (1918–20) and a non‐pandemic period (1917, 1921–25). Binomial logistic regression models were fit to predict the likelihood of a P&I death against four other major causes of death, first using only BMI as a predictor, then controlling for demographic variables. BMI alone can predict the probability of P&I death, but only during the pandemic period (*p* < 0.001). BMI (ORs = 0.90–1.51), all regions (ORs = 2.08–9.17), age (OR = 0.98), sex (male: OR = 0.66–0.75), and ethnicity group (non‐Alaska Native: OR = 0.36–0.37) significantly predicted the likelihood of a P&I death during the pandemic. The results suggest that as BMI increases, the risk of P&I death also increases with additional predictors, but only during the 1918 influenza pandemic period. There is no significant relationship between BMI and P&I death outside of pandemic years. This result may contribute an additional unique feature to our understanding of the 1918 influenza pandemic and its epidemiological novelty. This research further contributes new data on historical population biology and contextualizes results within the framework of developmental origins of health and disease for ultimate explanations of differential risks between Alaska Native and settler populations.

## 1 Introduction

Recently, there has been considerable advancement in our knowledge of unequal outcomes during the 1918 influenza pandemic (e.g., D'Adamo et al. 2023; Dimka et al. 2022; Mamelund and Dimka 2021) and epidemiological patterns in previously unstudied locations (e.g., Cavert 2022; Fourie and Jayes 2021; Nygaard et al. 2023; Sharma et al. 2021). Higher BMI was first identified as a potential risk factor for severe influenza and pneumonia (P&I) outcomes in 2009 during the H1N1 influenza A pandemic (Honce and Schultz‐Cherry 2019). Height and mass were typically not recorded on individual historical death records from the early 20th century outside of some military records (e.g., Noymer 2009; Summers et al. 2014). Aside from knowledge obtained through military data, the relationship between body size and the risk of severe 1918 flu outcomes is poorly understood in general, likely due to the scarcity of historical biometric data and gaps in our current knowledge about the nature of the immune response to the then‐novel H1N1 influenza A virus.

This paper uses historical biometric data (height and mass) recorded in individual death records from Alaska to investigate how body mass index (BMI), along with other demographic variables, contributed to the probability of a P&I death both during the 1918 flu (1918–1920) and in the immediately preceding and succeeding years (1917, 1921–1925). First, we discuss the proximate physiological research that has been conducted since 2009 to better understand how malnutrition and overnutrition may incur risks of severe outcomes due to infection with the influenza virus. Then, we discuss the developmental origins of health and disease as an ultimate evolutionary framework for the phenomena observed. Finally, we discuss the context of early 20th century Alaska and describe results of the analysis of the relationship between BMI and risk of death from P&I compared to the four other major causes of death during the two time periods (pandemic and non‐pandemic). Although BMI is not a comprehensive measure of the overall health of an individual, relationships have been observed between severe risks of hospitalization and death from influenza and both very low (Moser et al. 2018) and very high BMI (Louie et al. 2011; Moser et al. 2018). As such, the recording and survival of biometric data to the present day provide an opportunity for insight into historical population biology and whether contemporarily observed relationships between BMI and flu outcomes apply to the 1918 flu pandemic in Alaska.

### 1.1 Proximate Explanations: Nutritional and Immunopathological Factors

The historical data available to assess the relationship between body size and P&I outcomes is limited, but this relationship can be assessed in relatively more detail with contemporary body size, immunological, and pathological data. The immunological pathways to mitigate the disease process and facilitate recovery from an influenza virus are variable by body size, and the insight that both overnutrition *and* undernutrition have strong bearings on an individual's ability to resist, fight, and recover from influenza infection is not new (Flanigan and Sprunt 1956; Underwood 1789). Here, we outline physiological explanations for increased risk from severe influenza outcomes under variable conditions.

The impact of malnutrition on disease outcomes during the 1918 flu is most clearly demonstrated by the Indian experience. Regions experiencing famine during 1918 showed the most pronounced mortality due to influenza (Mills 1986). Other sources have similarly linked malnutrition and famine to pandemic flu outcomes in Mexico (Alexander 2019), Egypt (Rose 2021), Iran (Afkhami 2003), and Italy (Gaeta et al. 2020). The specific means by which undernutrition contributed to severe disease and death during the 1918 flu remain undetermined; however, studies have determined that low BMI is correlated with influenza incidence and severity in human influenza viral infections generally (Blumentals et al. 2011; Morgan et al. 2010; Moser et al. 2018; Okubo et al. 2017; Papadimitriou‐Olivgeris et al. 2020; Thangaraj et al. 2023). This heightened susceptibility among undernourished individuals is hypothesized to be due to altered nutrient absorption, chronic inflammation, and changes in metabolic, hormonal, microbiota, and glucoregulatory mechanisms (Hashimoto et al. 2012; Lesourd 1997; Qin et al. 2015; Savino et al. 2022).

Specifically, protein energy malnutrition (PEM) has been shown to increase mortality, increase viral titers in lung tissue, impair viral clearance, reduce inflammatory cell recruitment to the lungs, and decrease virus‐specific antibody and nucleoprotein‐specific CD8+ T cells in mice infected with influenza A virus (Taylor et al. 2013). It has been proposed that protein deficiency leads to defective antigen processing, as well as altered proliferation, function, and survival of antigen‐specific T cells (Conzen and Janeway 1988; Lesourd 1997; Nájera et al. 2004; Shaobin and Petro 1997; Taylor et al. 2013).

Alternatively, micronutrient undernutrition may predispose individuals to infection through a lack of cofactors involved in enzymes critical to immune function. Vitamin A is involved in the regulation of T cell proliferation (Kiss et al. 2008), monocyte and dendritic cell differentiation (Klebanoff et al. 2013; Vellozo et al. 2017), and the regulation of immunoglobulin expression (Gangopadhyay et al. 1996; Stephensen et al. 1996). This deficiency has been shown to increase proinflammatory responses, delay viral clearance, and increase susceptibility to secondary bacterial infections after influenza A virus infection (Penkert et al. 2021). Like vitamin A, vitamin C is involved in T cell proliferation and immunoglobulin production (Mousavi et al. 2019), as well as neutrophil chemotaxis (Elste et al. 2017). In murine influenza A virus infection, vitamin C deficiency is associated with increased inflammatory cell infiltration and damage to the lung (Kim et al. 2016; Li et al. 2006). Vitamin D has been implicated in the activation of antiviral innate immune gene transcription (Hannsdottir et al. 2008; Telcian et al. 2017) and the regulation of T cell responses (Jeffery et al. 2012). Vitamin E is protective against reactive oxygen species (Traber 2007) and is associated with many innate and acquired immune responses, including increased immunoglobulin levels and NK cell activity (Lee and Han 2018). This review highlights the many possible pathways for elevated risk of severe influenza outcomes due to undernutrition alone, regardless of body size.

As stated above, obesity (≥30 kg/m^2^) and morbid obesity (≥40 kg/m^2^) were independent risk factors for severe disease and death during the 2009 H1N1 influenza pandemic (Cocoros et al. 2013; Louie et al. 2011; Morgan et al. 2010; Van Kerkhove et al. 2011). Numerous mechanisms have been implicated in elevated risk, including increased cardiovascular damage (Siegers et al. 2020), attenuated wound repair (O'Brien et al. 2012), impairment of innate and adaptive immune responses (Blaszczak et al. 2021; Green and Beck 2017), and a chronic proinflammatory state (Christ et al. 2019; Hulme et al. 2021). Specifically, an increase in adiposity is associated with an increase in blood volume, pericardial‐ and perivascular‐specific adipose tissue, arterial pressure, coronary obstruction, and tachyarrhythmias (Lopez‐Jiminez et al. 2022). Variation in wound healing due to obesity has been linked to reductions in vascularity and alterations in immune responses (Gealekman et al. 2011). Increased adiposity is associated with variation in cytokine and chemokine profiles. Proinflammatory molecules such as TNFɑ (Easterbrook et al. 2011; Jung et al. 2013), CCL5 (Khan et al. 2014; Warren et al. 2015), CXCL2, CXCL10, and CCL7 (Khan et al. 2014) were shown to have an increased plasma concentration at baseline in obese mice. Interestingly, during influenza virus infection, these molecules all show a delayed increase in expression in mice models of obesity, suggesting that an increased proinflammatory baseline is also associated with a slow response to infectious stimuli (Honce and Schultz‐Cherry 2019; Smith et al. 2007; Warren et al. 2015).

Animal models of infection with the 1918 H1N1 virus indicate that immunopathology was particularly severe during the pandemic (de Wit et al. 2018; Kash et al. 2006; Kobasa et al. 2004, 2007; Memoli et al. 2009). It has been postulated that the severe lung injury seen at autopsy of individuals who died of viral pneumonia in 1918–1919 was induced by a disproportionate proinflammatory response to the virus (Jung et al. 2013; Xiao et al. 2013). It is possible that an increase in cardiovascular damage, impairment of wound repair, and the steady proinflammatory state of obese individuals may have compounded this immunopathology during the 1918 flu, leading to an increase in severe disease and death. Individuals with greater adiposity may have a higher baseline of inflammation, leading to greater increases in immunopathology compared to lean individuals.

Lastly, secondary bacterial pneumonia has been implicated as a major cause of death during the 1918 flu (Brundage and Shanks 2008; Chien et al. 2009; Morens et al. 2008). Obese individuals have a higher prevalence of lower respiratory tract infections in general (Maccioni et al. 2018; Pugliese et al. 2022). Obesity has been implicated as a risk factor for severe secondary bacterial pneumonia after influenza viral infection (Karlsson et al. 2013). We suggest that obese individuals would have been more vulnerable to secondary bacterial pneumonia after acute infection with the 1918 flu virus (H1N1 influenza A), leading to worse outcomes. There is less evidence for malnutrition contributing to increased risk of secondary bacterial pneumonia; however, studies have shown that undernutrition is associated with increased severity of bacterial pneumonia (Chisti et al. 2009; Viasus et al. 2022). Malnutrition is also a leading risk factor for TB (Carwile et al. 2022). Perhaps the most notable symptom of TB is wasting, leading to a bi‐directional association with undernutrition (Schwenk and Macallan 2000). TB was a strong determinant of increased mortality during the 1918 flu (Mamelund and Dimka 2019; Oei and Nishiura 2012; van Doren and Sattenspiel 2021). Although no causal mechanism has been identified, there are hypotheses related to active (interaction of pathogens and disease processes) and passive (overlap of age groups of highest mortality of TB and P&I) selection (Noymer 2009). It is possible that body size, especially wasting caused by TB disease, is part of this story.

We recognize the limitations to what BMI can measure and its interpretation. BMI is widely used to determine public health policies (Centers for Disease Control and Prevention 2024; Nuttall 2015; World Health Organization 2010). However, BMI is criticized for its inability to adequately consider the body composition of the individual (adiposity), making it a poor measure for understanding overall health (Burkhauser and Cawley 2008). BMI alone certainly cannot give insight into the complexities of undernutrition, immunological suppression, or the effects of secondary bacterial infection without considerable additional information. Further, growth studies using BMI are often used as predictors of adult health outcomes (e.g., Eriksson et al. 2001; Toschke et al. 2007), making this measure a deeply embedded determinant of how we understand the relationship between BMI and health. The lack of ability of the singular measure of obesity to predict adiposity, combined with the substantial public health interventions to “prevent” obesity (Walls et al. 2011), can lead to mischaracterization of health in large groups of people, like athletes (Garrido‐Chamorro et al. 2009; Kruschitz et al. 2013) and cold‐adapted populations (Ocobock and Niclou 2022; Ocobock et al. 2022). However, it is possible to engage with the measure of BMI while simultaneously remaining cognizant of its implications and limitations (Gutin 2018), especially while carefully accounting for historical and syndemic contexts of the topic at hand. In this paper, we use BMI as a proxy for body size to estimate the relationship between body size and risk of P&I death compared to other major causes of death in the early 20th century with these limitations in mind.

### 1.2 Ultimate Explanations: Developmental and Syndemic Factors

Above, we reviewed some physiological pathways through which influenza infection and flu disease severity may be variable depending on body size, nutritional level, and adiposity. These physiological mechanisms, however, are strongly based in more ultimate evolutionary explanations that biocultural and medical anthropologists have been studying for decades to better understand health inequalities. The specific context of the current study—early 20th century Alaska—is one defined by a long history of colonialism and massive societal changes, which may not only be an important determinant of 1918 flu experiences there but also of the overall population health of Alaska Native Peoples.

At the end of the 20th century, Barker and colleagues (Barker 1990, 1994; Barker et al. 1989, 1991) published a series of theoretical and empirical research correlating the fetal environment with adult health, specifically infant birthweight with the risk of cardiovascular disease, diabetes, and stroke. Barker (1990) suggested that attention to the intrauterine environment was important because there are critical times during development in which physiology may become permanently altered, known as periods of developmental plasticity (Finch and Crimmins 2004). This plasticity allows developing biology the flexibility to adjust trajectories of development to best match the environment to which they will be born (Gluckman et al. 2009). Even though this plasticity can have short‐term benefits for both the mother and the developing fetus, it can incur long‐term fitness costs on the individual once they are born and develop in an environment dissimilar from the one for which they prepared (Gluckman et al. 2005). These are termed *mismatches*, and when there are significant social and environmental changes between conception and adulthood, disease risk can be elevated (Gluckman et al. 2009).

Building on the concept of the fetal origins of adult disease, researchers began to develop a better understanding of how health is impacted not just by the intrauterine environment, but also by the context of development over an expanded timeframe, including early life and even the health and socioeconomic contexts of previous generations. The developmental origins of health and disease (DOHaD) framework covers all this temporal depth and highlights primary proximate mechanisms by which past generations, the fetal environment, and early life can drive the predictive adaptive response, and ultimately, adult health risks and health inequalities (Gluckman and Hanson 2006; Gluckman et al. 2010).

DOHaD is relevant to the current study because of the ultimate determinants of variable baseline health between Alaska Native Peoples and settlers in the early 20th century. Alaska Native Peoples today have a life expectancy at birth 5–7 years lower than the national US average, a modest burden of cardiovascular disease, hypertension, and Type 2 diabetes, a high burden of obesity, modestly elevated infectious disease burdens, and high rates of alcoholism, suicide, and accidents (Snodgrass 2013). This health profile is like that of at least a century ago and covered thoroughly by writings on regularly circulating pathogens, tuberculosis, and the severe impacts of cultural loss (e.g., Fortuine 1989, 2005; Williams 2009). DOHaD can trace ultimate determinants of these health inequalities to experiences with historical trauma and cultural loss, which can have biological and psychosocial impacts not only on the people who experienced these traumas directly but on those who descended from afflicted populations, as well.

Conching and Thayer (2019) outline two possible pathways through which cultural loss and trauma can lead to observable health consequences. First, directly experienced trauma can lead to epigenetic modifications that can impact immune regulation (Paccaud et al. 2015), nervous system responses (Uddin et al. 2013), and anxiety and fear (controlled by amygdala activity) (Swartz et al. 2016). Even *perceived* discrimination and marginalization can produce higher stress hormone levels (Thayer and Kuzawa 2011), contribute to high blood pressure (Dolezsar et al. 2014), and lead to obesity (Cozier et al. 2014). Second, epigenetic modifications that contribute to these outcomes are heritable. Severe maternal stress during pregnancy can lead to epigenetic methylation of the *NR3C1* promoter, whose job is to modulate the expression of the hypothalamic–pituitary–adrenal axis (the primary regulator of stress response) (Thayer et al. 2018), which has been linked to lower offspring birthweight (Mulligan et al. 2012), higher childhood adiposity, and higher adult BMI (Cao‐Lei et al. 2015). High cortisol levels in breastmilk are also linked to heightened fear reactivity (Nolvi et al. 2017), temperament (Jonas et al. 2015), and BMI (Hahn‐Holbrook et al. 2016). Conching and Thayer (2019) also astutely point out that maternal stress, poverty, and minority status are all common conditions for people who have experienced historical trauma, so these are compounding conditions that contribute to the intergenerational transmission of poor baseline health status.

Recently, van Doren (2025) has suggested that novel pandemic events may help create inequalities in the following decades stemming from intrauterine and postnatal development stressors. However, sociocultural and ecological stressors may in fact contribute to unequal experiences in the first place. To reunite this discussion with infectious diseases and the 1918 flu, we suggest that historical trauma and cultural loss led to biological stress in Alaska Native Peoples that contributed to lower baseline health status, including increased adiposity, reduced immune regulation, chronic inflammation, and nutritional stress. As such, drawing upon a popular concept in medical anthropology, we suggest that these historical stressors acted as important syndemic conditions to raise the risk of severe disease and mortality during the 1918 flu. *Syndemics* are generally conceptualized as the co‐infection with two or more co‐circulating pathogens that accelerate their mutual disease processes and amplify negative consequences (e.g., Singer and Clair 2003). However, and importantly, the definition of a syndemic has always included the clustering of adverse health conditions *and* social conditions (e.g., Singer's early studies of the SAVA syndemic: substance abuse, violence, and AIDS: Singer 1994, 1996). As such, this paper utilizes data to address one piece of this syndemic, explained by the intergenerational transmission of the consequences of colonialism: BMI as a potential risk factor for mortality during the 1918 flu.

### 1.3 The 1918 Influenza Pandemic in Alaska

The 1918 flu is considered one of the worst infectious disease outbreaks in history, with an estimated death count ranging from 15 to 40 million, or even up to 50–100 million individuals worldwide (about 2%–2.5% mortality) (Johnson and Mueller 2002; Patterson and Pyle 1991; Spreeuwenberg et al. 2018). The 1918 flu was nearly ubiquitous worldwide, and while its global spread is often attributed to the movement of troops during World War I, non‐belligerent nations also suffered from the deadly influenza outbreak in the fall and winter of 1918 in the northern hemisphere (Humphries 2013; Oxford et al. 2002). We focus our study on body size and risk of 1918 flu death in Alaska, then a territory of the US, which experienced particularly striking mortality during the pandemic. In 1918, Alaska reported 1672 excess deaths per 100 000 individuals compared to the previous year's mortality estimates (Avila and Topol 2018). This figure is nearly double the excess deaths reported by the entire US in 1918 (Luk et al. 2001). While worldwide mortality estimates averaged around 2.5%, Alaska regional mortality averages were much higher and more diverse, ranging from ~1%–38% mortality (Mamelund et al. 2013). Overall, Alaska Native individuals accounted for 83% of all recorded deaths from 1918 to 1920 (Sattenspiel et al. 2024).

**FIGURE 1:**
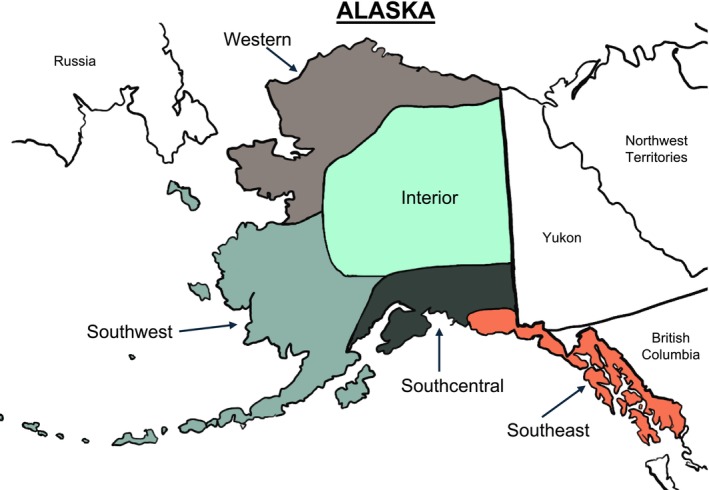
A map of Alaska with the five major regions identified: (1) Southeast = orange; (2) Western = brown; (3) Southcentral = dark green; (4) Southwest = sage green; (5) Interior = bright green. These regions were drawn from resources on the major cultural areas by statewide Alaska Native leadership (i.e., www.alaskanative.net).

Many sociodemographic and ecological factors may have contributed to the heterogeneous severity of the 1918 flu in Alaska (Crosby 1989; Mamelund 2011; Mamelund et al. 2013; Philip and Lackman 1962; Sattenspiel and Mamelund 2013; van Doren et al. 2023). In the early 20th century, the over 650 000 mile^2^ of territory was punctuated by rugged landscapes, unreliable food sources, and sub‐Arctic temperatures. Inadequate public infrastructure in much of the territory made land travel difficult, and sub‐zero temperatures limited river navigation for much of the year (Department of Commerce and Labor Bureau of the Census 1910). The vast territory can be split into five distinct regions for analyses: (1) Southeast Alaska, which includes Juneau and many remote island communities; (2) Western Alaska, which includes the North Slope, the Seward Peninsula, and everything north of the Arctic Circle; (3) Southcentral, which includes Anchorage and surrounding relatively well‐connected communities; (4) Southwest, which includes the Bristol Bay region and Aleutian Islands; and (5) the Interior, which is mountainous and difficult to access at some points of the year due to frozen rivers, ice, and snow. A map with these five regions can be found in Figure 1. This diversity impacted both the spread and severity of the pandemic, as populations were affected by known contributors of mortality heterogeneity, including environmental conditions, access to food and healthcare, public health responses and policies, and unequal distribution of government funding (Mamelund et al. 2013).

Many Alaska Native cultural groups practiced subsistence living, and groups traveled with food sources to establish seasonal settlements in the territory (Mamelund et al. 2013). For example, the Athabascan people moved from duck and muskrat camps in the spring to fishing camps in the summer, then finally to hunting sites in the winter (Crowell 2010). The Tlingit and Haida of Tlingít Aaní (Southeast Alaska) were dependent on fishing as well as hunting and gathering (especially berries) and migrated through mountainous regions in pursuit of seasonal food sources (Department of Commerce and Labor Bureau of the Census 1910; Goddard 1924). One notable exception to the nomadic patterns of Alaska Native groups was the Yup'ik/Sup'ik Peoples living around Bristol Bay. Due to a relatively warm sub‐Arctic climate and abundant natural resources, Yup'ik/Sup'ik Peoples practiced a less nomadic lifestyle compared to other groups (Spinney 2018). In contrast, many settlers migrated in and out of the territory with seasonal employment. Workers in the mining industry often left the territory in the fall and returned in the spring after water navigation became feasible again (Department of Commerce and Labor Bureau of the Census 1910). The interactions between migratory Alaska Native groups, more sedentary Alaska Native communities, and highly mobile non‐Alaska Native workers likely contributed to the transmission dynamics of the pandemic both within and outside of Alaska.

In this paper, we explore the relationship between BMI and the probability that, out of five major causes of death in the early 20th century in Alaska, a death during the 1918 pandemic period would be a P&I death. Given the complex nature of how malnutrition, undernutrition, and obesity relate to severe flu outcomes contemporarily, we hypothesize that, using BMI as a continuous variable with and without additional sociodemographic predictors, both low (underweight, ≤ 18.5 kg/m^2^) and high (morbid obesity, ≥ 40 kg/m^2^) BMI will result in increased risk of P&I death during the pandemic. We do not use these values as specific cut‐offs in our analyses but rather suspect we will see general trends that lend themselves to the complexity of the entwined determinants of nutritional status, developmental factors, and colonial histories between Alaska Native and non‐Alaska Native individuals.

## 2 Materials & Methods

### 2.1 Data Description

The primary data source used in this paper is the individual death records of people who died in Alaska from 1917 to 1925, obtained from the Alaska Health Analytics and Vital Records Section of the Alaska Bureau of Vital Statistics. Individual death records include data on location of death, name(s) of the deceased, age, sex, occupation, birthplace, primary and (less often) secondary causes of death, burial location, and anthropometrics such as height and mass. In these records, height was recorded in feet and inches while mass was recorded in pounds and ounces; conversions to meters and kilograms were made to calculate BMI for analyses using the standard equation (BMI = mass (kg)/height (m)^2^). We acknowledge there are limitations to the available data on anthropometrics in vital records, and these will be addressed in the discussion.

This research assesses recorded deaths from five major causes of death: (1) influenza and pneumonia (P&I); (2) diseases of the circulatory system; (3) diseases of the nervous system; (4) tuberculosis (TB); and (5) violence and accidents. Diseases of the circulatory and nervous systems were two of the leading categories of death in early 20th century Alaska, and together account for hypertension, arteriosclerosis, aneurysms, strokes, and other nonspecific paralytic diseases. Basic descriptions of the data can be found in Table 1. Overall, for the period of study, there were more males (68.9% during the pandemic period and 76.3% outside the pandemic), and there were roughly the same number of Alaska Native and non‐Alaska Native individuals during the pandemic period (51% and 49%, respectively), though non‐Alaska Native deaths accounted for 64.4% of the sample in the non‐pandemic period. Further, the Southeast had the largest number of death counts during the pandemic and in the surrounding years (28.6% and 37.6% of the sample, respectively), and the cause of death with the highest number of deaths during the pandemic was, unsurprisingly, P&I (44.3% of the sample), whereas outside of the pandemic it was violence and accidents (29.1%), though this is closely followed by TB (28.2%). TB was the leading cause of infectious disease death in Alaska for two centuries (Fortuine 2005).

**TABLE 1:** Description of data used in logistic regression analyses in this paper.

Variable	Pandemic	Non‐pandemic
Year		
1917	—	281 (19.6)
1918	670 (51.9)	—
1919	260 (20.2)	—
1920	360 (27.9)	—
1921	—	207 (14.4)
1922	—	196 (13.7)
1923	—	235 (16.4)
1924	—	257 (17.9)
1925	—	258 (18.0)
Sex		
Male	889 (68.9)	1094 (76.3)
Female	401 (31.1)	340 (23.7)
Ethnicity group		
Alaska Native	658 (51.0)	511 (35.6)
Non‐Alaska Native	632 (49.0)	923 (64.4)
District		
1: Southeast	368 (28.6)	530 (37.0)
2: Western	317 (24.5)	121 (8.4)
3: Southcentral	225 (17.5)	280 (19.5)
4: Southwest	222 (17.2)	275 (19.2)
5: Interior	158 (12.2)	206 (14.4)
Cause of death		
P&I	571 (44.3)	132 (9.2)
Tuberculosis	227 (17.5)	405 (28.2)
Circulatory	118 (9.2)	285 (19.9)
Nervous	60 (4.7)	194 (13.5)
Violence & accidents	314 (24.4)	418 (29.1)

*Note:* Individual death record counts are presented, along with the percent share of the sample each element represents (e.g., during the pandemic period, 51.9% of the sample is from 1918).

The violence and accidents category is included here not only because it was a major cause of death in early 20th century Alaska, but also to act as an informal point of reference based on its status as a non‐chronic and non‐infectious disease cause of death. We do not assume individuals who died of violence and accidents were healthier than those who died of other causes; there are no data available to substantiate this assumption. Violent and accidental deaths in early 20th century Alaska were most often drownings, hard labor accidents (mining, logging, and fishing), suicides, and homicides. As such, these causes of death occurred relatively quickly compared to chronic and infectious disease progressions, and the underlying health of the individual who suffered a violent or accidental death likely had less bearing on their cause of death. The analyses performed stratify the population by the five‐region structure illustrated in Figure 1. Finally, the sample was limited to those whose recorded age at death was ≥ 18 years. This was done to limit the number of individuals in the sample who may have still been growing to their adult size; while this is not a perfect assumption, this age limitation mitigates that bias. In total, the sample is *n* = 2724 individual death records. All analyses were performed in R version 4.3.1 with base R and tidyverse. De‐identified data are available with this publication in the Supporting Information, and the code will be available on the first author's GitHub page upon publication (https://www.github.com/tmvandoren). This project was presented to the Research Review Committee at the Alaska Native Tribal Health Consortium for early feedback.

### 2.2 Descriptive Analyses

Summary statistics were calculated for each of the five causes of death described above, including the minimum, median, mean, and maximum BMI for each cause of death in the complete sample. A visualization of yearly BMI distributions stratified by cause of death was generated. Density plots were generated of the BMI distributions for Alaska Native and non‐Alaska Native individuals stratified by major cause of death. Summary statistics for BMI distributions of Alaska Native and non‐Alaska Native subsets were calculated, and differences between means were tested for statistically significance with a two‐sided *t‐*test, assessed for significance at the *α* = 0.05 level.

### 2.3 Logistic Regression Analyses

A series of logistic regression models was the primary analytical method used to predict the probability that a death in the sample would be a P&I death (coded as 1) or one of the other causes of death (aggregately coded as 0). For each variation of logistic models, one was fit to the pandemic period (1918–1920) and one to the non‐pandemic period (1917, 1921–1925).

In the first set of logistic models, the probability of a P&I death was assessed using only BMI as a predictor. Assessing how to fit the BMI predictor in these models required consideration of the risks related to severe outcomes during P&I disease as a function of body size. We drew upon previously published knowledge related to P&I outcomes and risk, as outlined in the background section, and hypothesized that the relationship between BMI and the probability of P&I death may not be best represented by a linear relationship. As such, we additionally fit the baseline logistic regression model (using only BMI as a predictor) with quadratic and cubic transformations of the continuous BMI variable. Therefore, three baseline models were fit, as represented by Equations ((1), (2), (3)). The resultant coefficient estimates were exponentiated for interpretation of the odds ratio (OR) of each control variable. The quadratic and cubic models were optimized to identify the maxima and minima of the fitted models (i.e., the BMI where the probability of P&I death changes direction).

(1) $$\log\left(\frac{p\left(x\right)}{1-p\left(x\right)}\right)=\beta_{0}+\beta_{1}x_{\mathrm{BMI}}+\varepsilon$$

(2) $$\log\left(\frac{p\left(x\right)}{1-p\left(x\right)}\right)=\beta_{0}+\beta_{1}x_{\mathrm{BMI}}+\beta_{2}x_{\mathrm{BMI}}^{2}+\varepsilon$$

(3) $$\log\left(\frac{p\left(x\right)}{1-p\left(x\right)}\right)=\beta_{0}+\beta_{1}x_{\mathrm{BMI}}+\beta_{2}x_{\mathrm{BMI}}^{2}+\beta_{3}x_{\mathrm{BMI}}^{3}+\varepsilon$$

In the second set of logistic models, the probability of P&I death was assessed while controlling for BMI, region, sex, and ethnicity group. Like the baseline analysis, three models were fit to the data, one with linear, quadratic, and cubic transformations on BMI (Equations (4), (5), (6)). For these models, BMI is again a continuous variable. Region is a categorical variable that takes the following values: when *x* = 0, the region is Southeast; when *x* = 1, the region is Western; when *x* = 2, the region is Southcentral; when *x* = 3, the region is Southwest; and when *x* = 4, the region is Interior. Sex is a categorical variable where female = 0 and male = 1; age is a continuous variable describing age at death in years; and ethnicity is a categorical variable where Alaska Native = 0 and non‐Alaska Native = 1. For all logistic regression outputs, the estimated coefficients were exponentiated to obtain associated ORs. The quadratic and cubic multivariate models were optimized to identify where the probability of P&I death changes direction.

(4) $$\log\left(\frac{p\left(x\right)}{1-p\left(x\right)}\right)=\beta_{0}+\beta_{1}x_{\mathrm{BMI}}+\beta_{2}x_{\text{region}}+\beta_{3}x_{\mathrm{sex}}+\beta_{4}x_{\mathrm{age}}+\beta_{5}x_{\text{ethnicity}}+\varepsilon$$

(5) $$\begin{array}{c} \log\left(\frac{p\left(x\right)}{1-p\left(x\right)}\right)=\beta_{0}+\beta_{1}x_{\mathrm{BMI}}+\beta_{2}x_{\mathrm{BMI}}^{2}+\beta_{3}x_{\text{region}}\\ \;+\beta_{4}x_{\mathrm{sex}}+\beta_{5}x_{\mathrm{age}}+\beta_{6}x_{\text{ethnicity}}+\varepsilon \end{array}$$

(6) $$\begin{array}{c} \log\left(\frac{p\left(x\right)}{1-p\left(x\right)}\right)=\beta_{0}+\beta_{1}x_{\mathrm{BMI}}+\beta_{2}x_{\mathrm{BMI}}^{2}+\beta_{3}x_{\mathrm{BMI}}^{3}\\ \;+\beta_{4}x_{\text{region}}+\beta_{5}x_{\mathrm{sex}}+\beta_{6}x_{\mathrm{age}}\\ \;+\beta_{7}x_{\text{ethnicity}}+\varepsilon \end{array}$$

Finally, since logistic regression does not include *R* ^2^ values like ordinary least squares regression, we calculated McFadden's pseudo‐*R* ^2^ values, which are considered good analogs to adjusted‐*R* ^2^ values (Smith and McKenna 2013). This metric, also referred to as the likelihood ratio index, compares the relationship between the log likelihood of the model with full parameters to the log likelihood of the null model, which is represented by the intercept only. McFadden (1974) suggested that pseudo‐*R* ^2^ values between 0.2 and 0.4 are adequate values for model fit, so we use this guidance when assessing the relative fits for each of the models.

## 3 Results

### 3.1 Descriptive Results

**FIGURE 2:**
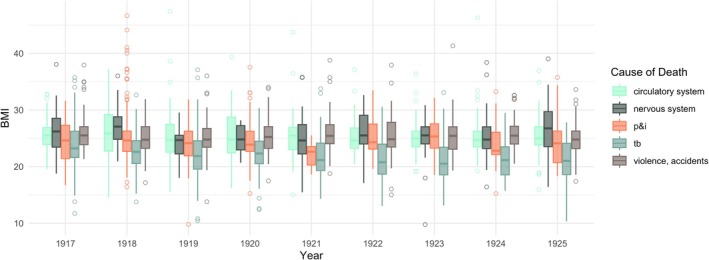
Boxplots of BMI for each cause of death by year for 1917–1925.

The summary statistics of the BMI distributions of all five major causes of death are reported in Table 2. Mean and median BMI at death are lowest for those who died of TB (21.9 and 22.0 kg/m^2^, respectively), potentially indicating wasting associated with long‐term affliction with the disease. This observation will not be expanded upon in this paper but will be pursued in further research. Mean and median BMI at death for the other four causes are similar to one another; mean BMI at death for violence and accidents, circulatory system causes, nervous system causes, and P&I are 25.4, 25.5, 25.6, and 24.7 kg/m^2^, respectively. Figure 2 illustrates these cause‐specific BMI distributions for each year of the study period (1917–1925).

**TABLE 2:** Summary statistics of minimum, median, mean, and maximum BMI for each of the five major causes of death analyzed in this paper.

Statistic	BMI by cause of death (kg/m^2^)					Ethnicity	
	Violence, accidents	Circulatory system	Nervous system	P&I	TB	Alaska Native	Non‐AN
Min	13.8	14.6	9.8	15.2	10.4	10.4	9.8
Median	25.1	24.8	25.5	24.3	21.9	23.3	24.8
Mean	25.4	25.5	25.6	24.7	22.0	23.6	25.2
Max	41.3	47.4	39.0	46.7	37.1	46.3	47.4

*Note:* Additionally, summary statistics stratified by ethnicity group are presented.

**FIGURE 3:**
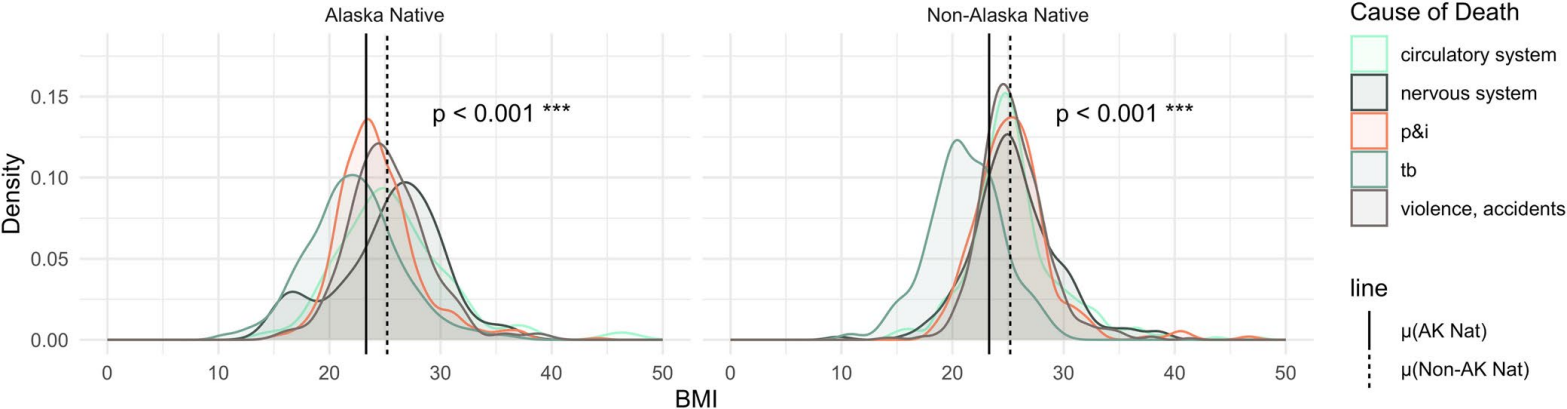
Density plots of BMI for Alaska Native and non‐Alaska Native individuals stratified by cause of death. Mean BMI at death for Alaska Native and non‐Alaska Native individuals is indicated by the solid and dashed lines, respectively; the solid and dashed lines that indicate mean BMI stratified by ethnicity are the same on each facet of the figure. The *p*‐value associated with the *t‐*test for significant differences is also indicated.

Table 2 also reports the descriptive statistics of BMI distributions for Alaska Native and non‐Alaska Native subsets of the primary dataset. Density plots of ethnicity‐based BMI at death stratified by major cause of death are in Figure 3. The mean BMI at death for Alaska Native and non‐Alaska Native groups is indicated by the solid and dashed vertical lines, respectively, where mean Alaska Native BMI was 23.6 kg/m^2^ and mean non‐Alaska Native BMI was 25.2 kg/m^2^. The difference in means was statistically significant at the *p* < 0.001 level.

### 3.2 Baseline Logistic Regression Results

The first set of logistic regression models tested the relationship between the probability of P&I death and BMI while only controlling for linear, quadratic, and cubic transformations of BMI. The results are presented in Table 3. BMI can only predict the probability of a P&I death during the 1918 flu with either a quadratic or cubic transformation of BMI. During the pandemic, the logistic model predicts that increases in BMI significantly increase the probability of P&I death up to the maximum, calculated as 29.0 kg/m^2^ (OR = 1.27, *p* = 0.016). Beyond the maximum, further increases in BMI decrease the risk of P&I death by 1% per unit increase in BMI (OR = 0.99, *p* = 0.028). McFadden's pseudo‐R^2^ signifies weak predictive power (0.0044). The pandemic model with the cubic transformation of BMI estimates significant fits for all parameters (all *p* < 0.001), but McFadden's pseudo‐R^2^ again suggests weak association (0.014), indicating that this model explains only 1.4% of the variation observed. During the non‐pandemic period, these relationships do not hold, and there is no statistical relationship between BMI and probability of death from P&I, regardless of polynomial transformations on BMI.

**TABLE 3:** Results of the logistic regression model using linear, quadratic, and cubic transformations of BMI to predict the probability of P&I death.

Time period	Linear		Quadratic		Vertices (kg/m^2^)	Cubic		Vertices (kg/m^2^)
	OR (error)	*p*	OR (error)	*p*		OR (error)	*p*	
Pandemic								
BMI	1.02 (1.01)	0.12	1.27 (1.10)	0.016*	Max = 29.0	10.6 (1.85)	< 0.001***	Max = 24.4
BMI^2^			0.99 (1.00)	0.028*		0.92 (1.02)	< 0.001***	Min = 35.2
BMI^3^						1.00 (1.00)	< 0.001***	
McFadden's p*R* ^2^	0.0014		0.0044			0.014		
Non‐pandemic								
BMI	1.00 (1.02)	0.86	1.17 (1.18)	0.36	Max = 23.4	1.90 (1.98)	0.35	Max = 23.6
BMI^2^			0.99 (1.00)	0.37		0.98 (1.03)	0.39	Min = 37.4
BMI^3^						1.00 (1.00)	0.44	
McFadden's p*R* ^2^	0.00012		0.0011			0.0017		

*Note:* Odds ratios (OR), their associated *p*‐values, vertices, and McFadden's pseudo‐*R* ^2^ are reported for each model. *p‐*value codes: *p* < 0.001 = ***; *p* < 0.01 = **; *p* < 0.05 = *; *p* < 0.10 = (.).

### 3.3 Multivariate Logistic Regression Results

**FIGURE 4:**
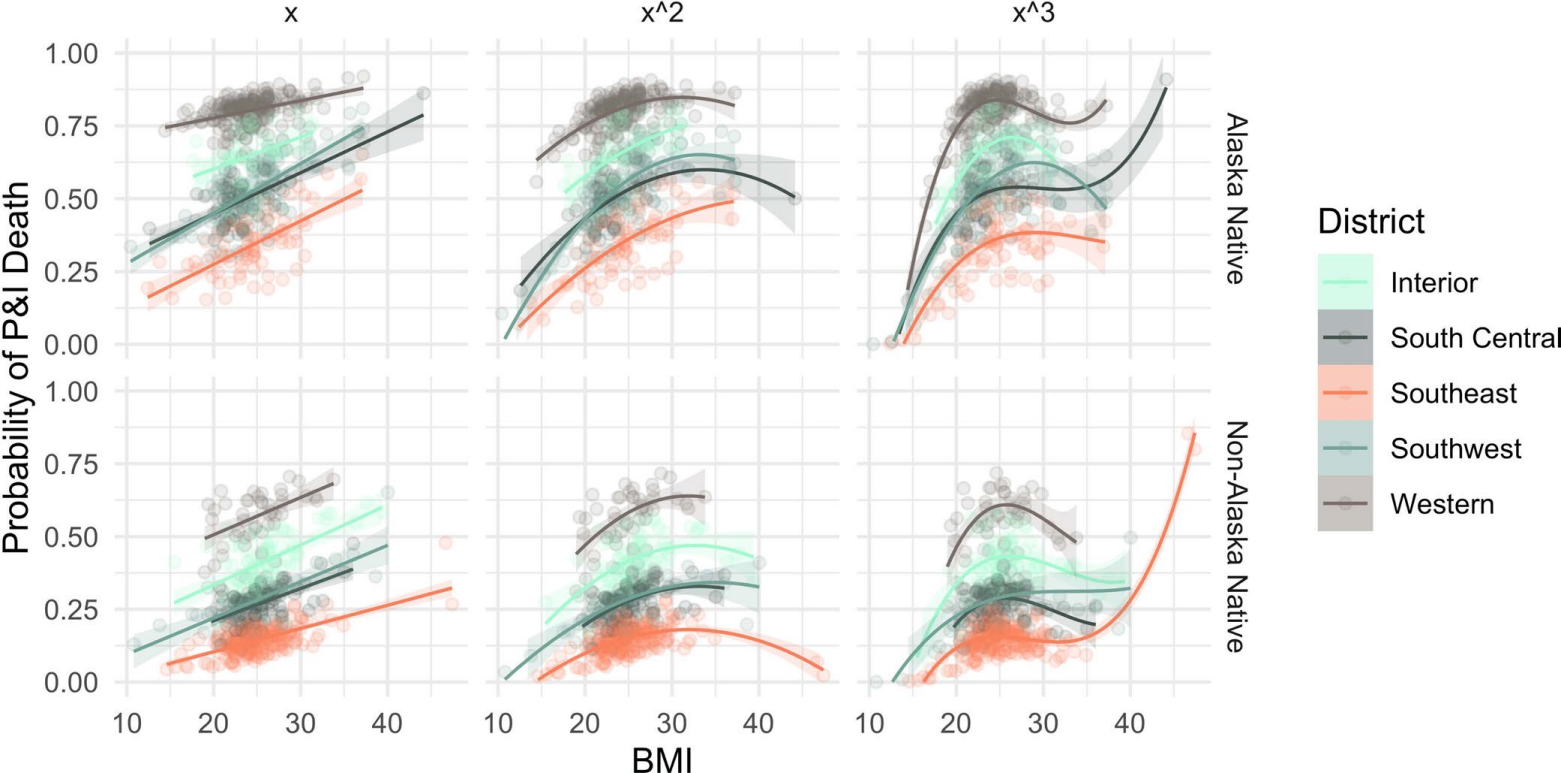
Predicted values of the linear (x), quadratic (x^2^), and cubic (x^3^) models for the probability of P&I death during the 1918 influenza pandemic period stratified by ethnicity and district.

**FIGURE 5:**
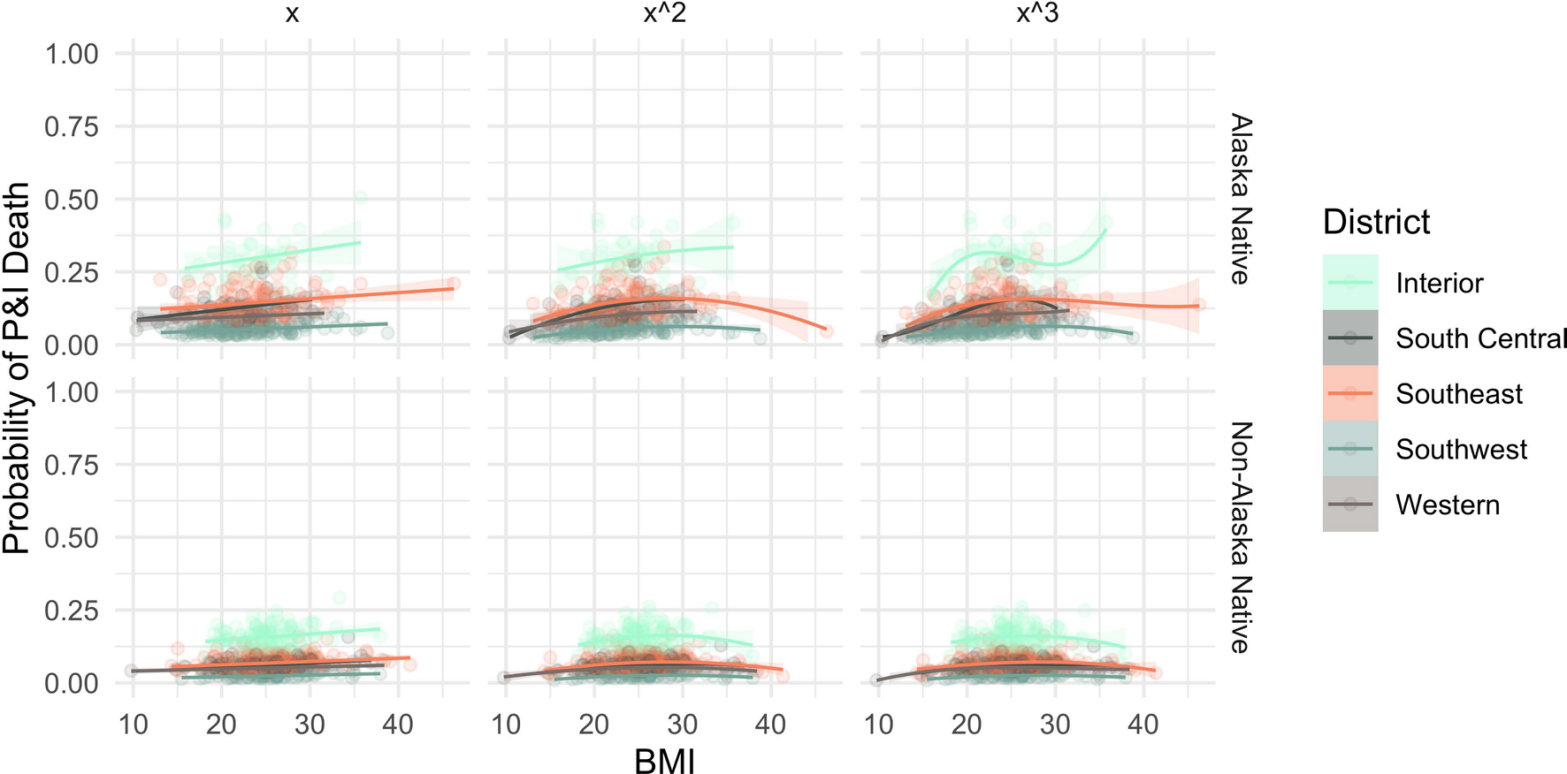
Predicted values for the linear (x), quadratic (x^2^), and cubic (x^3^) models for the probability of P&I death during the non‐pandemic period stratified by ethnicity and district.

The second set of logistic regression models tested the relationship between the probability of P&I death while controlling for district, age, sex, and ethnicity group. The relationships among BMI and the control variables are illustrated in Figures 4 and 5. Regression results, including ORs, *p*‐values, minimum and maximum values of the curves, and McFadden's pseudo‐R^2^ for the linear, quadratic, and cubic models for the pandemic and non‐pandemic periods are reported in Table 4.

**TABLE 4:** Results of the multivariate logistic regression models using linear, quadratic, and cubic transformations of BMI, district, age, sex, and ethnicity group to predict the probability of P&I death during the 1918 flu pandemic and outside of the pandemic.

	Linear	Quadratic	Vertices (kg/m^2^)	Cubic	Vertices (kg/m^2^)
	OR (error)	OR (error)		OR (error)	
Pandemic (1918–1920)					
BMI	1.07 (1.02)***	1.51 (1.13)***	Max = 30.5	21.1 (1.92)***	Max = 26.0
BMI^2^		0.99 (1.00)**		0.90 (1.02)***	Min = 35.3
BMI^3^				1.00 (1.00)***	
Region					
Southeast	Baseline				
Western	8.94 (1.23)***	8.88 (1.23)***		9.17 (1.23)***	
Southcentral	2.11 (1.22)***	2.11 (1.22)***		2.08 (1.23)***	
Southwest	2.25 (1.23)***	2.34 (1.23)***		2.50 (1.24)***	
Interior	4.39 (1.24)***	4.36 (1.24)***		4.46 (1.25)***	
Age	0.98 (1.00)***	0.98 (1.00)***		0.98 (1.00)***	
Sex					
Female	Baseline				
Male	0.75 (1.16)*	0.70 (1.16)*		0.66 (1.16)**	
Ethnicity					
Alaska Native	Baseline				
Non‐Alaska					
Native	0.37 (1.17)***	0.36 (1.16)***		0.36 (1.17)***	
McFadden's p*R* ^2^	0.19	0.20		0.21	
Non‐pandemic (1917, 1921–1925)					
BMI	1.01 (1.02)	1.24 (1.21)	Max = 26.8	1.82 (2.06)	Max = 25.3
BMI^2^		0.99 (1.00)		0.98 (1.03)	Min = 42.3
BMI^3^				1.00 (1.00)	
Region					
Southeast	Baseline				
Western	0.70 (1.47)	0.72 (1.47)		0.72 (1.47)	
Southcentral	0.96 (1.32)	0.96 (1.33)		0.95 (1.33)	
Southwest	0.37 (1.41)**	0.38 (1.41)**		0.38 (1.41)**	
Interior	2.42 (1.27)***	2.38 (1.27)**		2.37 (1.27)**	
Age	1.02 (1.01)**	1.02 (1.01)*		1.02 (1.01)*	
Sex					
Female	Baseline				
Male	0.74 (1.26)	0.70 (1.27)		0.70 (1.26)	
Ethnicity					
Alaska Native	Baseline				
Non‐Alaska					
Native	0.42 (1.26)***	0.41 (1.26)***		0.41 (1.26)***	
McFadden's p*R* ^2^	0.068	0.070		0.070	

*Note:* Odds ratios (OR), significance values, vertices, McFadden's pseudo‐*R* ^2^, and correct prediction percentage are reported for each model. *p*‐value codes: *p* < 0.001 = ***; *p* < 0.01 = **; *p* < 0.05 = *; *p* < 0.10 = (.).

The primary result of these analyses is that the probability of a P&I death compared to a non‐P&I death while controlling for BMI, district, age, sex, and ethnicity can only be confidently predicted during the 1918 flu pandemic period, and there is a significant increase in the probability of P&I death on the basis of BMI and district. Otherwise, outside of this major novel infectious disease event, there are few variables that can predict a P&I death during a non‐pandemic period. According to the calculated McFadden's pseudo‐*R* ^2^ values, all the models perform relatively the same within their study periods; the pandemic models range from pseudo‐*R* ^2^ values of 0.19–0.21, while the non‐pandemic models range from 0.068 to 0.070. The former range indicates decent fits, while the latter range indicates very poor fits.

Within the pandemic, BMI with any order transformation has strong predictive power for the probability of P&I death while controlling for all other variables. Aided by the visualization in Figure 4, there are general increases in the probability of P&I death per unit increase in BMI. Specifically, the linear model predicts a 7% higher probability of P&I death per unit increase in BMI (OR = 1.07, *p* < 0.001), the quadratic predicts a 51% higher probability per unit increase up to BMI 30.5 kg/m^2^ (OR = 1.51, *p* < 0.01), and the cubic predicts that those under 26.0 kg/m^2^ were over 21.1 times more likely to die from P&I during the pandemic (OR = 21.1, *p* < 0.01). This staggering increase could imply a true increase in the risk of P&I death with further increasing BMI, but given the small number of individuals with recorded BMI in this range, we consider this to be an indicator of overfitting the model with the cubic function. This will be discussed further. In addition to the increases observed for the quadratic and cubic polynomial fits, there are also significant decreases predicted for the probability of P&I death. The quadratic predicts a significant decline after BMI 30.5 kg/m^2^ (OR = 0.99, *p* < 0.05), while the cubic predicts a significant decline after BMI 26.0 kg/m^2^.

During the 1918 flu, region, age, sex, and ethnicity were also strong predictors of the probability of death for each of the models fit and tested. Because all ORs calculated for each variable and all transformations of BMI were similar in size and significance, we report their ranges rather than each individually. Compared to the baseline region (Southeast), the Western region had the highest probability of P&I death (OR = 8.88–9.17, all *p* < 0.001), followed by the Interior (OR = 4.36–4.46, all *p* < 0.001). Southcentral and Southwest Alaska had similar probabilities of P&I death during the pandemic to one another, but still significantly high odds compared to the Southeast (Southcentral: OR = 2.08–2.11, *p* < 0.001; Southwest: OR = 2.25–2.50, *p* < 0.001). Per‐unit increases in age also significantly predict a decrease in the probability of P&I death during the pandemic (all ORs = 0.98, *p* < 0.001). Males were significantly less likely to suffer a P&I death than females (ORs = 0.66–0.75, linear and quadratic *p* < 0.05, cubic *p* < 0.01). Finally, non‐Alaska Native individuals were significantly less likely to experience P&I death than Alaska Native individuals during the pandemic (ORs = 0.36–0.37, *p* < 0.001).

For the non‐pandemic period, there were no significant BMI predictors for the probability of P&I death even with controlling for other pertinent variables. In the non‐pandemic period, individuals in the Interior were significantly more likely to die from P&I than otherwise (ORs = 2.37–2.42, linear *p* < 0.001, quadratic and cubic *p* < 0.01), and there was a significantly lower probability of P&I death in the Southwest (ORs = 0.37–0.38, *p* < 0.01). All models predict a significantly lower probability of non‐Alaska Native P&I death (ORs = 0.41–0.42, all *p* < 0.001). Finally, all non‐pandemic models predicted significant increases in the probability of P&I death with per unit increases in age (all ORs = 1.02, linear *p* < 0.01, quadratic and cubic *p* < 0.05).

Briefly, for each model fit (using BMI only as well as BMI plus other control variables), we assessed the ratio of residual deviance over the degrees of freedom for additional understanding about whether our data were overdispersed or underdispersed, providing insight into the observed variance of the data and how well the coefficients are estimated. This check showed that the variance was overdispersed (more variance than expected) in all models that only used BMI as a predictor of probability of P&I death. Combined with the results presented in the previous section, specifically that the McFadden's pseudo‐*R* ^2^ values indicated relatively poor fits, there was too much variance in the data for these models to provide a reliable assessment of the relationships of interest. However, the additional predictors used in the multivariate models described here yielded ratios that indicate the data were not overdispersed; therefore, the multivariate models provide much more reliable estimates of the relationship between BMI, other demographic predictors, and the probability of P&I death.

## 4 Discussion

### 4.1 Historical Orientation of Results

The results presented in this study show that BMI is a significant predictor of the increased probability of P&I death during the 1918 influenza pandemic. In non‐pandemic years, there are other variables that can significantly predict P&I death, but BMI cannot. Further, BMI *alone* is not enough to confidently predict whether any given individual died from P&I or one of the other four aggregated causes. Rather, other variables such as region, sex, age, and ethnicity group must also be controlled to gain insight into how body size is related to the risk of P&I death.

In general, the logistic regression models for the pandemic show that the risk of P&I death increases up to BMI in the mid‐ to high‐20s kg/m^2^, and for Alaska Native individuals in all regions. Additionally, increases in age predict a small but significant decrease in the risk of P&I death. During the pandemic, males were significantly less likely to die from P&I. Finally, the probability of dying from P&I regardless of body size, region, age, sex, or ethnicity group was substantially higher during the 1918 flu than in non‐pandemic years (Figures 4 and 5). This result is not necessarily surprising but does present another way of interpreting the severity of the pandemic.

The substantially increased risk of P&I death for Alaska Native individuals in all regions, but especially in Western Alaska and the Interior, is not a surprising result in light of the original descriptions of the 1918 flu in the historical record. Worldwide, Indigenous Peoples are known to have experienced more severe outcomes during the pandemic than settler populations in the same regions (Mamelund 2003; Mamelund et al. 2013; Mølbak Ingholt et al. 2019; Nygaard et al. 2023; Rice 2018). This was especially true in Alaska, where remote areas of the Seward Peninsula (Western) suffered some of the highest mortality rates in the world, to the point where many villages' survivors were children, leading to a high burden of orphans and abandonment of settlements (Lauterat 1986; Mamelund et al. 2013). Mamelund (2011) suggests that the percent mortality in Teller, Nome, and Wales was 90%, 58.3%, and 54.8%, respectively. In total, the Governor of Alaska, Thomas Riggs, reported to US Congress that ~90% of pandemic deaths in Alaska were Alaska Native individuals, with an estimated 950 Alaska Native deaths in Nome alone (Sisson et al. 1919).

The results of this study support written historical evidence and other more recent research that Alaska Native individuals were at substantially higher risk of P&I death. This observation holds true for the non‐pandemic period as well. All multivariate models predict that non‐Alaska Native individuals have significantly less risk of death from P&I during the pandemic (OR = 0.38) *and* outside the pandemic (OR = 0.46). All multivariate regression results indicate that individuals in Western Alaska were almost nine times (> 900%) more likely to die of P&I during the pandemic than those in the Southeast. The other predicted ORs related to district were comparably smaller but still range from two times (> 200%) up to over four times (> 400%) increased risk compared to the Southeast. The sex‐based differences identified here, which show that males were less likely to die of P&I during the 1918 flu pandemic, join the ranks of variable findings on this topic worldwide, where there have been significantly more severe consequences observed in males (Garenne 2015; Noymer and Garenne 2000), in females (Mamelund et al. 2016; Tripp et al. 2018), as well as no clear differences at all (Bengtsson et al. 2018; Paskoff and Sattenspiel 2019; Tuckel et al. 2006; Viboud et al. 2013).

With respect to age, a characteristic that sets the 1918 flu apart from regularly circulating, seasonal flus is its widely observed “signature” age‐based pattern of mortality, where younger adults aged 15–40 experienced the highest excess mortality in almost every location studied (Luk et al. 2001). However, this was not a universal observation, as some rural Sami, Alaska Native, and Greenland Inuit had different age patterns of mortality (Ingholt et al. 2024; Mamelund 2011; Nygaard et al. 2023; Sattenspiel et al. 2024). Even though the multivariate models presented in this paper do control for both ethnicity group and age, they do not consider the different age patterns of mortality between Alaska Native and non‐Alaska Native groups, as this has been done previously (Sattenspiel et al. 2024).

The “signature” age‐based pattern of 1918 flu mortality only describes the non‐Alaska Native pattern, for which mortality in ages 25–29 peaked at 111.7 deaths per 10 000 (Sattenspiel et al. 2024). The Alaska Native age‐based pattern of 1918 flu mortality has high mortality among younger adults, but exhibits even higher mortality among those aged 35–49, with a peak in years 40–44 at 618 deaths per 10 000 (Sattenspiel et al. 2024). While Sattenspiel et al. (2024) do find decreasing age‐based mortality beyond these ages (specifically in 55–59), we cannot ignore the possibility that the detected reduction in risk of P&I death with increasing age may reflect a lower life expectancy, and therefore fewer deaths in general beyond age 60. Finally, we must also recognize that the present study only considers individuals who died at age 18 or older; P&I is a common cause of infant and young child illness and death in typical non‐pandemic years, so the relationship between children and risk of death from P&I regardless of body size would likely yield separate relationships.

Interpreting the relationship between body size and risk of P&I death is difficult, as there are many factors that could contribute to overall risk. BMI does not consider body proportions or composition of adipose tissue to muscle tissue, making it a poor marker of overall health status. Further, populations adapted to cold climates tend to have higher BMI due to ecological pressures of lower average temperature (Katzmarzyk and Leonard 1998), while emerging biocultural anthropological research suggests that there are potentially metabolically healthy obese phenotypes in Arctic‐adapted Indigenous Peoples (Hopping et al. 2010; Ocobock and Niclou 2022; Ocobock et al. 2022). As a population, Inuit have one of the highest average BMIs and a high proportion of overweight and obese individuals, yet relatively lower blood pressure and other markers of health like plasma insulin and triglycerides (Galloway et al. 2000; Young et al. 2007). Further, in the background section, we discussed the complexity of protein energy malnutrition, micronutrition, undernutrition, and immunopathologies associated with each, and we want to re‐emphasize that the physiology underlying susceptibility to severe influenza disease is not as simple as BMI. We caution against interpretations that uncritically associate higher BMI with poor health, especially in cold climate adapted populations which exhibit cold adapted anatomy (higher adiposity, fat free mass, overall body size, and BMI), cold adapted physiology (higher resting metabolic rates in brown adipose tissue), high activity levels leading to higher cardiovascular fitness, and consumption of high protein and low carbohydrate diets (Ocobock and Niclou 2022).

Despite this, the BMI distributions calculated in this paper show that in this sample, Alaska Native individuals had significantly *lower* BMI at death than non‐Alaska Native individuals (23.3 kg/m^2^ vs. 25.1 kg/m^2^, respectively, *p* < 0.001) (Figure 3, Table 2). This phenomenon may not necessarily be contradictory to the body of anthropological research that parses the relationship between body size and latitude or temperature. Rather, it could be reflective of the embodiment of childhood nutritional stressors (Bjerregaard et al. 2014; Druet and Ong 2008), a heavy burden of TB, which can lead to wasting (Fortuine 2005; Martin and Sabina 2018; Paton and Ng 2006), and the lasting effects of the history of bi‐directional colonialism (from Russia and the U.S.) that severed Alaska Native people from their cultures (Czyzewski 2011; Poppel 2017). This discrepancy speaks strongly to the dynamic nature of population biology over a century, and that contemporary knowledge of body size and composition may not be an accurate reflection of that of only a century ago.

The immunopathologies associated with protein energy malnutrition, micronutrition, and undernutrition described are proximate explanations for how individuals with varying levels of nutrition—not necessarily body size—fight influenza infection. However, the conditions that contribute to health disparities between Indigenous Peoples and non‐Indigenous individuals in the same locations may be contextualized with ultimate explanations of the embodiment of historical trauma and cultural loss through the lenses of DOHaD and syndemic effects. Land dispossession, displacement, and nutritional disruption are non‐mutually exclusive determinants that can afflict significant stress on the body, and epigenetic modifications in response to those stressors can be passed down intergenerationally. Contemporary research with Indigenous youth has shown that even today, individuals who regularly thought about cultural and land dispossession had significantly higher levels of depression and anxiety than those who did not, even if they had not directly experienced the trauma their ancestors did (Altaha and Kraus 2017; Armenta et al. 2016).

Before and during the early 20th century in Alaska, displacement and dispossession of lands and waters, in addition to forcible movement of communities, are significant features of colonial impact. This would have had a strong direct impact on Alaska Native communities, which rely heavily on subsistence hunting and gathering of nutritionally dense animals and plants (Redwood et al. 2008). A decrease in nutrient and diet diversity, as well as the chronic stress of displacement and dispossession, can have severe consequences on long‐term health on their own. However, the DOHaD framework may additionally suggest that proximate nutritional stressors can instigate epigenetic modifications in the affected individual, which may be inherited in future generations. Thus, there is likely a complex evolutionary push‐and‐pull, whereby high latitude physiology provides relatively strong adaptations to the environment while extant colonial factors cause reductions in fitness. This mismatch may have shaped the unequal susceptibility to the novel and acute 1918 influenza pandemic virus observed in Alaska Native Peoples.

### 4.2 Interpretation of Model Variations

Here, we briefly discuss how contemporary knowledge of the relationship between BMI and the risk of severe influenza outcomes aligns with how we chose to model this relationship and the results it produced. First, McFadden's pseudo‐*R* ^2^ values assessed between pandemic and non‐pandemic models clearly show that the variables selected can predict P&I death more accurately during the pandemic (Tables 3 and 4). This could be due to the substantial burden of P&I deaths during the pandemic, while the non‐pandemic period can be characterized better by a substantial burden of TB (Fortuine 1989, 2005), as well as a high representation of violent and accidental deaths. Again, the ability of the models to predict significantly higher probability of a P&I death during the 1918 flu pandemic is not a surprising result, but the primary result of interest is the significant relationship between increasing BMI and the elevated risk of P&I death during the pandemic *only*.

Within the set of pandemic models, it is more difficult to assess which model (linear, quadratic, or cubic transformation of BMI) best represents the relationship between controls and risk of P&I death. Given the similarities among McFadden's pseudo‐*R* ^2^ values, each of the three suggests relatively good fit and predictive power. However, research on the relationship between BMI and contemporary influenza outcomes suggests that the relationship is not linear, but rather that there are higher risks for people under ~20 kg/m^2^ and over ~40 kg/m^2^ (Moser et al. 2018). These two high‐risk groups cover the risks associated with being underweight, particularly having fewer fat stores for energy use during illness (Dobner and Kaser 2018; Green et al. 2021), and of high adiposity, in which persistent pro‐inflammatory states exist that compromise immune function (Rojas‐Osornio et al. 2019). Thus, we did not assume a linear relationship for the historical data, and this body of work was a primary motivator to test higher‐order transformations of BMI in the prediction of P&I death.

Further, the motivation to fit the cubic transformation of BMI to predict the probability of P&I death was due to the substantially increased risk for those with BMI ≥ 40 kg/m^2^ to be hospitalized and/or die from P&I. However, the research that produced this result was carried out with contemporary data for which higher BMI is increasingly common. Given this repeatedly observed result, we sought to identify this relationship in the historical Alaskan data, as well. The density plots of BMI for all individuals in the data clearly show that there are very few people whose BMI at death was at or above 40 kg/m^2^ (Figure 3, Table 2). Specifically, there are only nine people in this sample with a BMI ≥ 40 kg/m^2^, and over half of them died of P&I during the 1918 flu. Compared to the proportions of individuals with slightly lower BMI (in the 35–39 kg/m^2^ range), this is an over‐representation of P&I deaths by BMI. We cautiously suggest that this result indicates an elevated risk of P&I death for individuals with BMI ≥ 40 kg/m^2^ during the 1918 influenza pandemic. Ultimately, although there were some individuals in the sample with higher ranges of BMI, the results of the cubic model fits (Table 4, Figures 4 and 5) suggest that there are too few points in this range to give insight into the relationship between very high BMI and increased risk of P&I death, even though contemporary influenza data provide more clear evidence of this relationship. This can be posited from the OR values predicted by the cubic model for the pandemic in Table 4: beyond the BMI value of 34.9 kg/m^2^, the OR for the remaining values is 1.00—indicating that there is neither an increase nor decrease in risk for the few BMI values above this minimum. Additionally, in the models that predicted the probability of P&I death *only* with BMI, the predicted values increased in significance with increasing power transformations, which is not necessarily a reliable indicator of improved model validity. Thus, for this population, the cubic model is likely overfit and is not a good reflection of the nature of this relationship. We conclude that linear and quadratic transformations of BMI for early 20th century Alaska for P&I and these other four primary causes of death may be appropriate for populations with BMI distributions that do not include a large proportion of the population in the ≥ 40 kg/m^2^ range. For contemporary Western populations where this would be more common (James 2004) and for which overfitting a cubic model would be less of a risk, a cubic model to capture increased risk in two very different ranges of body size may be more appropriate.

### 4.3 Limitations

There are several limitations of this research that we must acknowledge. Primarily, the height and mass measurements that appear on death records are height and mass *at death*. There are two reasons this is an important consideration for the interpretation of any result. First, many individuals suffered from infectious and/or chronic conditions for long periods of time before death, such as TB, circulatory diseases, and nutritional stress (Fortuine 2005). Many of these conditions can have a strong bearing on the mass of the afflicted individual, especially TB and nutritional disorders, which can cause wasting (Paton and Ng 2006; Schwenk and Macallan 2000). The death records do not account for the process of mass change through illness (i.e., the records do not contain multiple measurements over time for the same individual); therefore, height and mass at death can only capture the end of the process.

We acknowledge the possibility that the large number of TB deaths in the sample may have helped decrease the overall average body size of the sample, especially for Alaska Native individuals via the disease's chronic wasting process. However, in the absence of longitudinal data for individuals who died of TB (indeed, lack of longitudinal data for all individuals in the dataset), we cannot know for sure if wasting was prevalent, or to what extent. Additionally, by including four other major causes of death aside from P&I during this time period, we found the inclusion of TB deaths to be essential to approximate a reasonable representation of the population at the time. We do, however, believe it is significant that the probability of mortality controlling for BMI among the other variables was so elevated during the 1918 influenza pandemic compared to the other aggregate causes, in which TB deaths were part. This not only speaks to the severity of the pandemic's novelty, but to the unique immunopathological stressors caused by the novel influenza virus.

The second reason height and mass at death need to be interpreted with caution is because there are likely variations in accuracy between measurements recorded during autopsy and those that were not. It may be assumed that many heights—and especially body masses—were estimates because in the process of cleaning and doing initial descriptive analyses there was evidence of heaping. That is, mass was more likely to end with a “0” or a “5” as a rounding or estimation phenomenon rather than the recording of precise measurements. This is important to acknowledge, but descriptive analyses and density plots presented in this paper indicate that BMI was still relatively normally distributed around a feasible mean (Figure 2).

Another limitation is that the number of individuals with *both* height and mass recorded at death was relatively small compared to the total number of individuals that died in the period studied. A total of 5690 individuals died from one of the five causes of death considered in this paper from 1917 to 1925; therefore, the sample of 2724 records in this paper for whom BMI could be calculated accounts for nearly 48% of the total number of individuals who died from P&I, TB, cardiovascular, nervous, or violent/accidental causes. While this is not a prohibitive level of representation for this sample, we must also acknowledge that, in four of the hardest hit localities in Alaska (Port Clarence, Wales, Nome, and Teller in the Seward Peninsula of Western Alaska), there were 399 Alaska Native individuals recorded to have died from P&I during November–December 1918 that had neither height nor mass recorded. The presence of data from these individuals in the pandemic dataset could have increased the pandemic sample by up to 31%, which would have undoubtedly influenced the model predictions for risk of P&I death in the Western region. Despite the inability to account for individuals with missing data, the models still predicted that individuals in the Western region were over eight times more likely to suffer a P&I death during the pandemic compared to the baseline (Southeast) region (*p* < 0.001), so this substantial impact was nevertheless detected with strong predictive power. Finally, and in the same vein, we must acknowledge that there is likely a data collection bias based on geography and sociocultural factors. Of most importance is the probable undercounting of pandemic P&I deaths in remote villages of Alaska, especially for Alaska Native groups who are seasonally migratory (Howe 2009; Lewis et al. 2023), compared to regions with relatively larger settler populations, like in the Southeast (Juneau, Sitka) and Southcentral (Anchorage).

## 5 Conclusion

We conclude that, while there are indications that increasing BMI can predict a higher probability of P&I death during the 1918 flu in Alaska, there are other important sociodemographic factors that likely played a more significant role in the variable risk of P&I death during the pandemic compared to the other major causes of death observed during this time period. Additionally, the relationship between BMI and the probability of death *outside* of the 1918 flu is weak and cannot be improved with the inclusion of additional predictors. Overall, this research contributes insights into how height and mass, and therefore BMI, in a historical Arctic population may have contributed to 1918 flu outcomes. This is an important but otherwise poorly understood dimension of the historical pandemic's impacts in a non‐military population, especially in a region of the world (the Arctic) where historical data generally and biometric data specifically are relatively scarce.

## Ethics Statement

The authors have nothing to report.

## Conflicts of Interest

The authors declare no conflicts of interest.

## Supporting information


**Data S1:** ajhb70151‐sup‐0001‐DataS1.csv.

## Data Availability

The data that supports the findings of this study are available in the Supporting Information of this article.
